# Supplementary material for: Adaptive Seedling Strategies in Seasonally Dry Tropical Forests: A Comparative Study of Six Tree Species
Source: Plants (Basel). 2024 Oct 17;13(20):2900. doi: 10.3390/plants13202900 (PMC11510953; doi:10.3390/plants13202900)
Supplement: Supplementary file 1 [file plants-13-02900-s001.zip › plants-3220386-supplementary.pdf]

Species	Seed weight	Germination	Root/shoot ratio	Seedling growth rate
Acacia_macr	0.082	7.000	0.371	0.291
Acacia_macr	0.071	41.000	0.250	0.249
Acacia_macr	0.058	11.000	0.432	0.187
Acacia_macr	0.061	13.000	0.346	0.196
Acacia_macr	0.068	29.000	0.345	0.317
Acacia_macr	0.058	50.000	0.177	0.261
Acacia_macr	0.052	34.000	0.260	0.295
Acacia_macr	0.049	18.000	0.371	0.265
Centrolobium	36.249	21.000	0.209	0.149
Centrolobium	42.428	28.000	0.314	0.257
Centrolobium	15.002	16.000	0.210	0.252
Centrolobium	17.139	16.000	0.137	0.297
Centrolobium	22.547	21.000	0.283	0.188
Centrolobium	35.965	16.000	0.239	0.239
Centrolobium	30.021	16.000	0.297	0.167
Centrolobium	17.735	16.000	0.270	0.178
Centrolobium	36.863	16.000	0.198	0.151
Centrolobium	11.530	16.000	0.215	0.222
Centrolobium	29.110	28.000	0.356	0.317
Centrolobium	33.348	16.000	0.307	0.184
Centrolobium	29.653	16.000	0.358	0.162
Centrolobium	30.374	16.000	0.410	0.212
Centrolobium	28.982	21.000	0.254	0.282
Centrolobium	38.244	23.000	0.309	0.211
Centrolobium	13.975	30.000	0.225	0.294
Centrolobium	31.771	28.000	0.174	0.398
Centrolobium	7.302	21.000	0.183	0.258
Centrolobium	29.406	16.000	0.401	0.234
Centrolobium	15.022	16.000	0.402	0.140
Centrolobium	29.809	23.000	0.505	0.159
Centrolobium	25.143	21.000	0.332	0.321
Centrolobium	33.039	23.000	0.181	0.282
Centrolobium	25.950	23.000	0.399	0.275
Centrolobium	34.125	16.000	0.375	0.211
Centrolobium	28.405	16.000	0.433	0.189
Centrolobium	33.162	21.000	0.288	0.197
Centrolobium	25.311	16.000	0.511	0.289
Centrolobium	54.610	30.000	NA	0.101
Centrolobium	11.173	16.000	0.141	0.241
Centrolobium	16.944	19.000	0.296	0.247
Centrolobium	37.485	28.000	0.266	0.386
Centrolobium	19.075	28.000	0.154	0.215
Centrolobium	12.964	22.000	0.184	0.361
Centrolobium	30.009	29.000	0.224	0.256
Centrolobium	31.048	18.000	0.441	0.245
Centrolobium	30.800	27.000	0.116	0.277
Centrolobium	28.528	15.000	0.276	0.279
Centrolobium	10.040	27.000	0.125	0.322
Centrolobium	31.472	20.000	0.313	0.303
Centrolobium	20.093	25.000	0.274	0.300
Centrolobium	24.442	18.000	0.398	0.187
Centrolobium	18.179	27.000	0.193	0.258

Centrolobium	20.950	36.000	0.218	0.278
Centrolobium	24.803	15.000	0.333	0.247
Centrolobium	12.044	15.000	0.175	0.254
Centrolobium	19.914	27.000	0.189	0.308
Centrolobium	17.653	15.000	0.257	0.213
Centrolobium	20.896	27.000	0.444	0.284
Centrolobium	25.413	20.000	0.338	0.188
Centrolobium	18.089	18.000	0.343	0.259
Centrolobium	34.966	20.000	0.333	0.246
Centrolobium	28.562	27.000	0.318	0.418
Centrolobium	43.016	27.000	0.336	0.430
Centrolobium	41.704	15.000	0.272	0.232
Centrolobium	35.964	22.000	0.428	0.344
Centrolobium	15.212	20.000	0.385	0.304
Centrolobium	35.146	29.000	0.325	0.252
Centrolobium	27.649	22.000	0.704	0.283
Centrolobium	19.153	20.000	0.405	0.359
Centrolobium	17.504	36.000	0.161	0.333
Centrolobium	32.210	27.000	0.200	0.272
Centrolobium	11.194	18.000	0.463	0.322
Centrolobium	41.223	22.000	0.281	0.407
Centrolobium	15.169	12.000	0.344	0.230
Centrolobium	17.743	16.000	0.111	0.353
Centrolobium	30.917	16.000	0.272	0.401
Centrolobium	15.794	19.000	0.140	0.428
Centrolobium	31.653	19.000	0.165	0.458
Centrolobium	33.311	16.000	0.216	0.278
Centrolobium	16.914	12.000	0.147	0.242
Centrolobium	29.536	19.000	0.257	0.348
Centrolobium	28.724	12.000	0.360	0.324
Centrolobium	11.814	14.000	0.205	0.292
Centrolobium	35.418	14.000	0.121	0.290
Centrolobium	25.425	30.000	0.317	0.501
Centrolobium	32.211	16.000	0.187	0.329
Centrolobium	30.638	16.000	0.296	0.306
Centrolobium	39.014	16.000	0.348	0.363
Centrolobium	31.172	19.000	0.136	0.494
Centrolobium	31.085	21.000	0.204	0.556
Centrolobium	35.479	16.000	0.198	0.405
Centrolobium	16.516	19.000	0.160	0.375
Centrolobium	17.737	12.000	0.205	0.316
Centrolobium	16.972	19.000	0.134	0.362
Centrolobium	32.308	23.000	0.145	0.615
Centrolobium	21.629	14.000	0.180	0.297
Centrolobium	24.138	16.000	0.299	0.201
Centrolobium	27.633	14.000	0.214	0.366
Centrolobium	29.573	23.000	0.222	0.228
Centrolobium	24.019	35.000	0.179	0.272
Centrolobium	28.655	26.000	0.213	0.649
Centrolobium	31.463	26.000	0.320	0.455
Centrolobium	18.497	19.000	0.220	0.477
Centrolobium	13.376	21.000	0.184	0.468
Centrolobium	14.501	19.000	0.135	0.440

Centrolobium	23.175	26.000	0.257	0.511
Centrolobium	24.613	19.000	0.217	0.471
Centrolobium	20.085	19.000	0.281	0.327
Centrolobium	24.800	14.000	0.264	0.417
Centrolobium	17.549	14.000	0.186	0.302
Centrolobium	16.072	16.000	0.221	0.302
Centrolobium	25.700	23.000	0.123	0.442
Centrolobium	19.435	28.000	0.335	0.597
Centrolobium	10.265	28.000	0.148	0.666
Centrolobium	21.009	23.000	0.127	0.470
Centrolobium	43.672	15.000	0.320	0.373
Centrolobium	41.384	18.000	0.236	0.374
Centrolobium	24.525	25.000	0.147	0.348
Centrolobium	33.995	18.000	0.391	0.442
Centrolobium	28.393	22.000	0.160	0.393
Centrolobium	24.620	20.000	0.084	0.454
Centrolobium	33.346	22.000	0.300	0.594
Centrolobium	30.953	18.000	0.304	0.391
Centrolobium	17.080	20.000	0.176	0.551
Centrolobium	41.314	25.000	0.137	0.516
Centrolobium	44.696	15.000	0.280	0.466
Centrolobium	18.651	18.000	0.146	0.479
Centrolobium	37.291	27.000	0.250	0.627
Centrolobium	48.429	18.000	0.310	0.412
Centrolobium	27.709	13.000	0.149	0.346
Centrolobium	30.214	34.000	0.258	0.652
Centrolobium	33.903	18.000	0.286	0.456
Centrolobium	23.349	15.000	0.324	0.395
Centrolobium	36.360	18.000	0.179	0.445
Centrolobium	33.346	13.000	0.282	0.423
Centrolobium	44.665	18.000	0.186	0.533
Centrolobium	22.355	18.000	0.265	0.284
Centrolobium	25.040	18.000	0.204	0.392
Centrolobium	42.950	22.000	0.121	0.361
Centrolobium	30.630	27.000	0.162	0.454
Centrolobium	31.148	15.000	0.412	0.355
Centrolobium	10.165	18.000	0.250	0.446
Centrolobium	36.424	22.000	0.104	0.256
Centrolobium	50.044	15.000	0.252	0.356
Centrolobium	42.631	34.000	0.364	0.695
Centrolobium	33.381	20.000	0.238	0.409
Centrolobium	21.543	22.000	0.165	0.416
Centrolobium	23.794	20.000	0.270	0.379
Coccoloba_ri	0.046	9.000	0.264	0.150
Coccoloba_ri	0.040	5.000	0.176	0.176
Coccoloba_ri	0.044	5.000	0.346	0.122
Coccoloba_ri	0.043	5.000	0.420	0.126
Coccoloba_ri	0.049	12.000	0.220	0.105
Coccoloba_ri	0.047	5.000	0.294	0.128
Coccoloba_ri	0.043	12.000	0.201	0.144
Coccoloba_ri	0.045	12.000	0.293	0.119
Coccoloba_ri	0.045	5.000	0.213	0.156
Coccoloba_ri	0.044	16.000	0.124	0.115

Coccoloba_r	0.043	12.000	0.207	0.123
Coccoloba_r	0.040	12.000	0.289	0.125
Coccoloba_r	0.040	9.000	0.144	0.095
Coccoloba_r	0.038	9.000	0.213	0.137
Coccoloba_r	0.038	9.000	0.121	0.122
Coccoloba_r	0.052	5.000	0.135	0.128
Coccoloba_r	0.047	9.000	0.260	0.139
Coccoloba_r	0.045	9.000	0.162	0.151
Coccoloba_r	0.049	9.000	0.230	0.131
Coccoloba_r	0.040	5.000	0.210	0.125
Coccoloba_r	0.041	12.000	0.348	0.129
Cynophalla_	0.186	10.000	0.061	0.125
Cynophalla_	0.272	10.000	0.086	0.295
Cynophalla_	0.228	10.000	0.119	0.132
Cynophalla_	0.272	10.000	0.095	0.195
Cynophalla_	0.271	10.000	0.102	0.269
Cynophalla_	0.337	10.000	0.108	0.160
Cynophalla_	0.284	10.000	0.127	0.215
Cynophalla_	0.262	10.000	0.092	0.219
Cynophalla_	0.276	10.000	0.110	0.156
Cynophalla_	0.210	10.000	0.123	0.241
Cynophalla_	0.224	10.000	0.106	0.212
Cynophalla_	0.217	10.000	0.229	0.121
Cynophalla_	0.177	10.000	0.179	0.187
Cynophalla_	0.189	10.000	0.223	0.121
Cynophalla_	0.216	10.000	0.161	0.179
Cynophalla_	0.164	10.000	0.117	0.162
Cynophalla_	0.139	10.000	0.103	0.072
Cynophalla_	0.181	10.000	0.156	0.175
Cynophalla_	0.154	10.000	0.102	0.174
Cynophalla_	0.156	10.000	0.115	0.165
Cynophalla_	0.194	10.000	0.137	0.155
Cynophalla_	0.201	10.000	0.091	0.145
Cynophalla_	0.206	10.000	0.086	0.161
Cynophalla_	0.091	10.000	0.087	0.066
Cynophalla_	0.117	10.000	0.175	0.120
Cynophalla_	0.249	10.000	0.118	0.175
Cynophalla_	0.144	10.000	0.188	0.123
Cynophalla_	0.213	21.000	0.101	0.164
Cynophalla_	0.138	10.000	0.052	0.113
Cynophalla_	0.223	10.000	0.106	0.185
Cynophalla_	0.155	21.000	0.100	0.052
Cynophalla_	0.164	21.000	0.106	0.147
Cynophalla_	0.182	10.000	0.073	0.143
Cynophalla_	0.179	10.000	0.045	0.144
Cynophalla_	0.192	21.000	0.076	0.167
Cynophalla_	0.190	21.000	0.137	0.211
Cynophalla_	0.200	21.000	0.203	0.167
Cynophalla_	0.213	21.000	0.152	0.124
Cynophalla_	0.189	10.000	0.072	0.248
Cynophalla_	0.134	10.000	0.056	0.133
Cynophalla_	0.147	10.000	0.085	0.164
Cynophalla_	0.144	21.000	0.173	0.099

Cynophalla_ı	0.176	10.000	0.147	0.191
Cynophalla_ı	0.148	10.000	0.097	0.188
Cynophalla_ı	0.137	10.000	0.161	0.164
Cynophalla_ı	0.135	10.000	0.143	0.170
Cynophalla_ı	0.363	10.000	0.146	0.160
Cynophalla_ı	0.222	10.000	0.148	0.178
Cynophalla_ı	0.319	10.000	0.114	0.204
Cynophalla_ı	0.199	10.000	0.170	0.164
Cynophalla_ı	0.263	10.000	0.236	0.124
Cynophalla_ı	0.251	10.000	0.139	0.250
Cynophalla_ı	0.254	21.000	0.046	0.026
Cynophalla_ı	0.295	10.000	0.128	0.156
Cynophalla_ı	0.203	10.000	0.126	0.102
Cynophalla_ı	0.288	21.000	0.185	0.193
Cynophalla_ı	0.199	21.000	0.188	0.234
Cynophalla_ı	0.162	10.000	0.139	0.182
Cynophalla_ı	0.176	10.000	0.088	0.150
Cynophalla_ı	0.170	10.000	0.109	0.071
Cynophalla_ı	0.265	31.000	0.056	0.106
Cynophalla_ı	0.196	10.000	0.065	0.156
Cynophalla_ı	0.189	21.000	0.135	0.067
Cynophalla_ı	0.200	10.000	0.156	0.173
Cynophalla_ı	0.193	10.000	0.060	0.159
Cynophalla_ı	0.220	21.000	0.076	0.117
Cynophalla_ı	0.209	10.000	0.138	0.171
Cynophalla_ı	0.205	10.000	0.105	0.192
Cynophalla_ı	0.201	10.000	0.282	0.043
Cynophalla_ı	0.112	21.000	0.166	0.143
Cynophalla_ı	0.173	21.000	0.074	0.081
Cynophalla_ı	0.193	10.000	0.111	0.131
Cynophalla_ı	0.148	21.000	0.253	0.187
Cynophalla_ı	0.199	10.000	0.190	0.135
Cynophalla_ı	0.160	10.000	0.107	0.141
Cynophalla_ı	0.171	21.000	0.143	0.176
Cynophalla_ı	0.124	10.000	0.142	0.106
Cynophalla_ı	0.133	21.000	0.099	0.093
Cynophalla_ı	0.125	10.000	0.145	0.102
Cynophalla_ı	0.118	21.000	0.120	0.123
Cynophalla_ı	0.143	10.000	0.068	0.148
Cynophalla_ı	0.122	10.000	0.271	0.114
Cynophalla_ı	0.124	21.000	0.171	0.142
Cynophalla_ı	0.138	10.000	0.160	0.178
Cynophalla_ı	0.145	10.000	0.100	0.110
Cynophalla_ı	0.134	10.000	0.110	0.103
Cynophalla_ı	0.101	10.000	0.114	0.105
Erythrina_vel	0.323	4.000	0.629	0.096
Erythrina_vel	0.408	6.000	0.965	0.090
Erythrina_vel	0.354	6.000	0.875	0.061
Erythrina_vel	0.342	6.000	0.781	0.063
Erythrina_vel	0.313	4.000	0.685	0.085
Erythrina_vel	0.296	6.000	0.833	0.103
Erythrina_vel	0.296	151.000	0.110	0.059
Erythrina_vel	0.321	39.000	0.453	0.175

Erythrina_vel	0.267	5.000	0.465	0.131
Erythrina_vel	0.294	5.000	0.598	0.110
Erythrina_vel	0.268	5.000	0.494	0.153
Erythrina_vel	0.162	7.000	0.468	0.179
Erythrina_vel	0.281	12.000	0.375	0.167
Erythrina_vel	0.251	9.000	0.805	0.136
Erythrina_vel	0.257	5.000	0.345	0.141
Erythrina_vel	0.274	133.000	0.138	0.023
Erythrina_vel	0.276	68.000	0.222	0.185
Erythrina_vel	0.252	9.000	0.489	0.150
Erythrina_vel	0.274	9.000	0.568	0.138
Terminalia_v	0.683	115.000	0.500	0.073
Terminalia_v	0.868	94.000	0.524	0.158
Terminalia_v	1.202	87.000	0.700	0.183
Terminalia_v	0.668	75.000	0.419	0.169
Terminalia_v	0.734	101.000	0.438	0.182
Terminalia_v	0.683	115.000	0.125	0.073
Terminalia_v	0.608	59.000	0.852	0.125
Terminalia_v	1.196	75.000	0.591	0.178
Terminalia_v	1.085	59.000	0.895	0.074
Terminalia_v	1.154	70.000	0.667	0.163
Terminalia_v	0.825	66.000	0.833	0.127
Terminalia_v	1.260	66.000	0.875	0.106
Terminalia_v	1.151	115.000	0.357	0.132
Terminalia_v	0.844	75.000	0.370	0.134
Terminalia_v	0.566	75.000	0.345	0.175
Terminalia_v	0.663	115.000	0.400	0.111
Terminalia_v	0.612	115.000	0.088	0.125
Terminalia_v	0.811	75.000	0.619	0.136
Terminalia_v	0.884	63.000	0.167	0.058
Terminalia_v	0.785	56.000	0.769	0.089
Terminalia_v	1.083	42.000	0.571	0.037
Terminalia_v	1.036	75.000	0.652	0.127
Terminalia_v	0.857	80.000	0.591	0.162
Terminalia_v	1.154	66.000	0.462	0.105
Terminalia_v	1.024	80.000	0.765	0.137
Terminalia_v	1.086	45.000	0.500	0.050
Terminalia_v	0.703	59.000	0.850	0.088
Terminalia_v	0.734	59.000	0.833	0.101
Terminalia_v	0.562	66.000	0.529	0.117
Terminalia_v	0.637	45.000	1.095	0.034
Terminalia_v	0.732	66.000	0.850	0.140
Terminalia_v	0.661	56.000	0.930	0.105
Terminalia_v	0.649	94.000	0.167	0.115
Terminalia_v	0.699	52.000	0.762	0.083
Terminalia_v	0.667	59.000	0.667	0.078
Terminalia_v	0.582	52.000	0.759	0.116
Terminalia_v	0.695	66.000	0.684	0.179
Terminalia_v	0.643	86.000	0.703	0.155
Terminalia_v	0.818	96.000	0.208	0.140
Terminalia_v	0.573	86.000	1.926	0.166
Terminalia_v	0.746	68.000	0.721	0.122
Terminalia_v	0.968	86.000	0.589	0.146

Terminalia_v	0.570	68.000	0.637	0.110
Terminalia_v	0.650	54.000	0.635	0.059
Terminalia_v	0.686	119.000	0.198	0.102
Terminalia_v	0.531	119.000	0.361	0.078
Terminalia_v	0.735	119.000	0.900	0.011
